# Supplementary material for: The genome sequence of True’s beaked whale, Mesoplodon mirus True, 1913 (Artiodactyla: Ziphiidae)
Source: Wellcome Open Res. 2026 Jan 6;11:10. [Version 1] doi: 10.12688/wellcomeopenres.25413.1 (PMC12853023; doi:10.12688/wellcomeopenres.25413.1)
Supplement: Peer review history

# Reviewer report: Reviewer response for version 1

**DOI:** 10.21956/wellcomeopenres.27999.r144384

**Published:** 2026-01-28

**Authors:** Mariana F Nery

**Affiliations:** 1 State University of Campinas, São Paulo, Brazil

This manuscript presents a high-quality chromosomal-level genome assembly for *Mesoplodon mirus* as part of the Darwin Tree of Life Project. The work is technically sound, well-executed, and clearly written. The assembly metrics are excellent, and the methods are described in sufficient detail for reproducibility. 

 My only doubt is regarding the chromosome assignment percentage. 79,62% of the assembly is assigned to chromosomes, which falls short of the EBP general benchmark. What would comprise the remaining ~20%?

Are sufficient details of methods and materials provided to allow replication by others?

Yes

Is the rationale for creating the dataset(s) clearly described?

Yes

Are the datasets clearly presented in a useable and accessible format?

Yes

Are the protocols appropriate and is the work technically sound?

Yes

Reviewer Expertise:

Genomics

I confirm that I have read this submission and believe that I have an appropriate level of expertise to confirm that it is of an acceptable scientific standard.

# Reviewer report: Reviewer response for version 1

**DOI:** 10.21956/wellcomeopenres.27999.r144382

**Published:** 2026-01-19

**Authors:** Jayan Duminda M Senevirathna

**Affiliations:** 1 Uva Wellassa University, Badulla, Uva Province, Sri Lanka

The manuscript presents a well-executed and clearly written genome assembly for *Mesoplodon mirus*. The authors have produced a high-quality draft assembly supported by strong metrics, including a BUSCO completeness of 97.8%, which is fully acceptable for a vertebrate genome and indicates that the assembly captures the vast majority of expected conserved genes.

 The combination of adequate sequencing depth, appropriate assembly tools, and thorough contamination assessment (e.g., blob plot analysis) further supports the reliability of the assembly.

 The methods are described in sufficient detail to allow reproducibility, and the results are logically presented and well supported by figures and tables. The manuscript is also strengthened by clear data accessibility statements.

 Only minor improvements are suggested, such as adding clarification on annotation parameter settings and briefly discussing the small proportion of missing BUSCOs to provide context regarding potential assembly gaps or repetitive regions. With these minor revisions addressed, the manuscript is suitable for indexing.

Are sufficient details of methods and materials provided to allow replication by others?

Yes

Is the rationale for creating the dataset(s) clearly described?

Yes

Are the datasets clearly presented in a useable and accessible format?

Yes

Are the protocols appropriate and is the work technically sound?

Yes

Reviewer Expertise:

Aquatic Biotechnology and Genetics

I confirm that I have read this submission and believe that I have an appropriate level of expertise to confirm that it is of an acceptable scientific standard.
